# A novel podocyte gene, semaphorin 3G, protects glomerular podocyte from lipopolysaccharide-induced inflammation

**DOI:** 10.1038/srep25955

**Published:** 2016-05-16

**Authors:** Ryoichi Ishibashi, Minoru Takemoto, Yoshihiro Akimoto, Takahiro Ishikawa, Peng He, Yoshiro Maezawa, Kenichi Sakamoto, Yuya Tsurutani, Shintaro Ide, Kana Ide, Harukiyo Kawamura, Kazuki Kobayashi, Hirotake Tokuyama, Karl Tryggvason, Christer Betsholtz, Koutaro Yokote

**Affiliations:** 1 Department of Clinical Cell Biology and Medicine, Chiba University Graduate School of Medicine, Chiba, Japan; 2 Division of Diabetes, Metabolism and Endocrinology, Chiba University Hospital, Chiba, Japan; 3 Department of Anatomy, Kyorin University School of Medicine, Mitaka, Tokyo, Japan; 4 Yokohama Rosai hospital, Yokohama, Japan; 5 Eastern Chiba Medical Center, Chiba, Japan; 6 Yu-karigaoka Tokuyama Clinic, Chiba, Japan; 7 Cardiovascular and Metabolic Disorders Program, Duke-NUS Graduate Medical School, Singapore, Singapore; 8 Department of Immunology, Genetics and Pathology, Cancer and Vascular Biology, Uppsala Universitet, Uppsala, Sweden

## Abstract

Kidney diseases including diabetic nephropathy have become huge medical problems, although its precise mechanisms are still far from understood. In order to increase our knowledge about the patho-physiology of kidney, we have previously identified >300 kidney glomerulus-enriched transcripts through large-scale sequencing and microarray profiling of the mouse glomerular transcriptome. One of the glomerulus-specific transcripts identified was semaphorin 3G (Sema3G) which belongs to the semaphorin family. The aim of this study was to analyze both the *in vivo* and *in vitro* functions of Sema3G in the kidney. Sema3G was expressed in glomerular podocytes. Although Sema3G knockout mice did not show obvious glomerular defects, ultrastructural analyses revealed partially aberrant podocyte foot processes structures. When these mice were injected with lipopolysaccharide to induce acute inflammation or streptozotocin to induce diabetes, the lack of Sema3G resulted in increased albuminuria. The lack of Sema3G in podocytes also enhanced the expression of inflammatory cytokines including chemokine ligand 2 and interleukin 6. On the other hand, the presence of Sema3G attenuated their expression through the inhibition of lipopolysaccharide-induced Toll like receptor 4 signaling. Taken together, our results surmise that the Sema3G protein is secreted by podocytes and protects podocytes from inflammatory kidney diseases and diabetic nephropathy.

The prevalence of kidney disease has explosively increased worldwide, mainly due to the increase in the number of patients who suffer from diabetic nephropathy (DN)^1^. Although the treatment for DN is important to improve the patients’ prognosis, the current treatment remains suboptimal and therefore novel approaches for DN are urgently needed.

Most of the kidney diseases are initiated from injuries of the glomerulus. Among the cell types that comprise the glomerulus, the podocyte is important both physiologically and pathologically. Large-scale sequencing of glomerular transcripts and comprehensive transcriptional profiling using glomerular cDNA microarray have revealed several podocyte-specific genes^2^,^3^,^4^. Among them, we have identified a gene named semaphorin 3G (Sema3G), previously called “semaphorin, sem2”, as one of the podocyte-expressed genes. This gene belongs to a family of secreted, class 3 semaphorins. The protein encoded by semaphorin 3A (Sema3A) was the first semaphorin that was characterized as a chemo-repulsive agent in neuronal growth^5^ and later found to play a role in angiogenesis^6^. The biological functions of semaphorins are diverse and they are reportedly involved in cell motility, growth, differentiation, and apoptosis^7^,^8^. The major receptors for class 3 semaphorins are neuropilins and plexins^9^. However, the functions of Sema3G in glomerulus or podocytes are not known.

There is global evidence that acute and/or chronic inflammation triggers and enhances kidney disease^10^, we therefore analyzed the function of Sema3G *in vivo* and *in vitro*, especially focusing on inflammation.

## Results

### Sema3G was expressed in podocytes and a secreted protein

**Figure 1:**
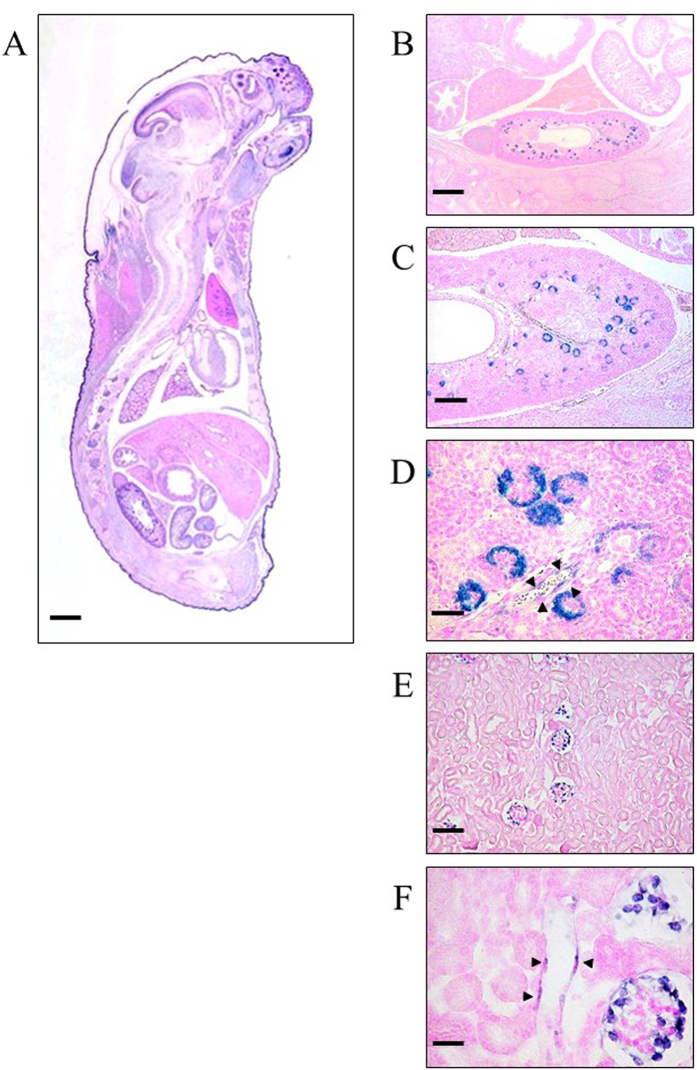
Sema3G is expressed in podocytes. *In situ hybridization* on E18.5 mouse embryo (**A–D**) and 4-month-old mice (**E**,**F**) revealed that Sema3G mRNA was expressed in developing podocytes and mature podocytes. Sema3G was expressed in endothelial cells in interlobular arteries outside the glomerulus (arrow heads indicated). Scale bars: 200 μm (**C**), 50μm (**D**), 100 μm (**E**), 25μm (**F**).

**Figure 2:**
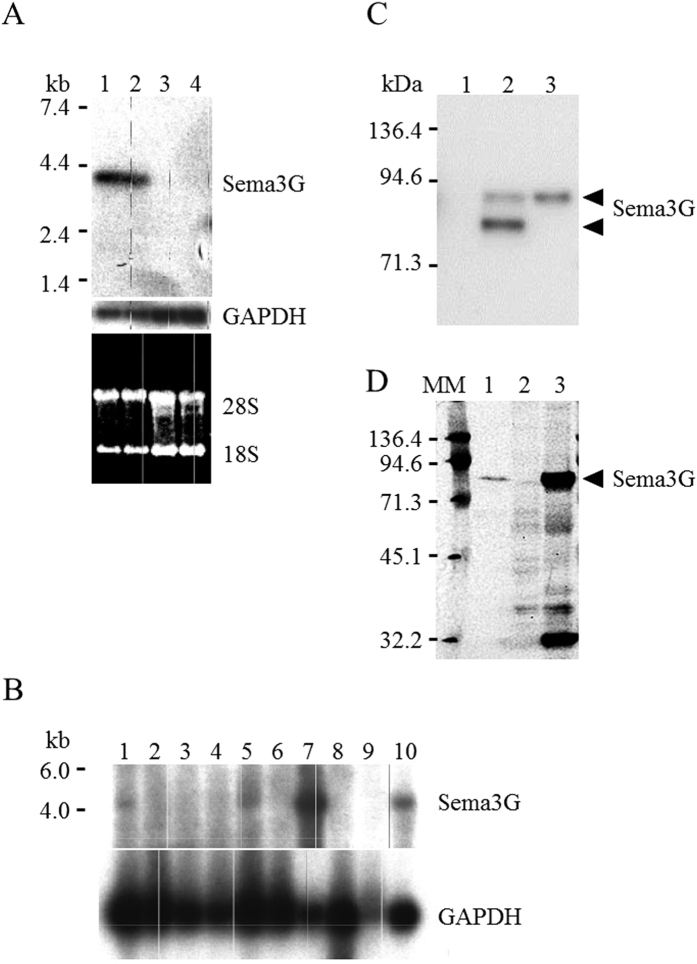
Sema3G was expressed in vascular-enriched tissues and secreted protein. (**A**) Northern blot analyses revealed Sema3G mRNA was highly expressed in the glomeruli. *Lane 1 and 2*: RNA from isolated glomeruli. *Lane 3 and 4:* RNA from whol^*−/−*^e kidney. (**B**) Tissue blot revealed that Sema3G was expressed in vascular-enriched tissues. *Lane 1: Heart, 2: Brain, 3: Liver, 4: Spleen, 5: Kidney, 6: Embryo (E14.0), 7: Lung, 8: Thymus, 9: Testis, 10: Ovary.* (**C**) Sema3G had stable expression in COS 7 cells and Sema3G protein expression was evaluated by Western blotting. *Lane 1: Cell lysate from control COS 7 cells. Lane 2: Cell lysate from Sema3G-stably-expressed COS 7 cells. Lane 3: conditioned medium from Sema3G-stably-expressed COS 7 cells.* (**D**) Sema3G protein expression was evaluated in the conditioned medium prepared from cultured human podocytes by Western blotting. *Lane 1:* Conditioned medium prepared from cultured human podocytes*. Lane 2:* Cell lysate prepared from cultured human podocytes. *Lane 3: Cell lysate from Sema3G-stably-expressed COS 7 cells. MM: molecular markers.*

First, we examined the expression of Sema3G on mouse embryonic day 18.5 (E18.5) and 4-month-old mice by non-radioactive *in situ* hybridization. We localized the Sema3G mRNA expression to glomerular podocytes (Fig. 1). During development, the expression started in the S-shaped body, continuing through the capillary loop stages and throughout the mature podocytes. Sema3G was also constitutively expressed in adult podocytes. We also detected Sema3G in endothelial cells in interlobular renal arteries not only in E18.5 embryos but also in adult mice. The sense probes produced no signals (Supplemental Figure 1). Because the expression of Sema3G was also observed outside the glomeruli, we performed northern blot analyses on different organs including isolated glomeruli. These revealed that the highest expression of Sema3G was in the lung tissue followed by kidney, heart, and ovary, indicating that Sema3G is expressed in vascular-enriched organs (Fig. 2A,B).

Based on its protein structure, it has been speculated that Sema3G belongs to a family of secreted, class-3 semaphorins. Sema3G was consistently expressed in COS 7 cells using retroviral systems. Then, western blotting using anti-Sema3G specific antibody revealed that Sema3G protein was expressed in both the cytoplasm and the conditioned medium showing a 94-kDa protein size, but not in the medium containing the control cells (Fig. 2C). We also confirmed that Sema3G was secreted from human cultured podocytes (Fig. 2D). These results confirmed that the Sema3G protein was a secreted protein. It has been reported that Sema3G bound to neuropilin 1 and/or neuropilin 2 for transducing intracellular signaling. The expression of neuropilin 1 and neuropilin 2 was examined, and it was found that the neuropilin 2 was not expressed within the glomeruli (unpublished observation); however, neuropilin 1 was expressed in all cell types within the glomeruli. Therefore, Sema3G, which was secreted from podocytes, might give signals not only to endothelial and mesangial cells but also to podocytes in a paracrine and/or autocrine manner (Supplemental Figure 3).

### Sema3G is important for podocyte morphology and overall kidney function

**Figure 3:**
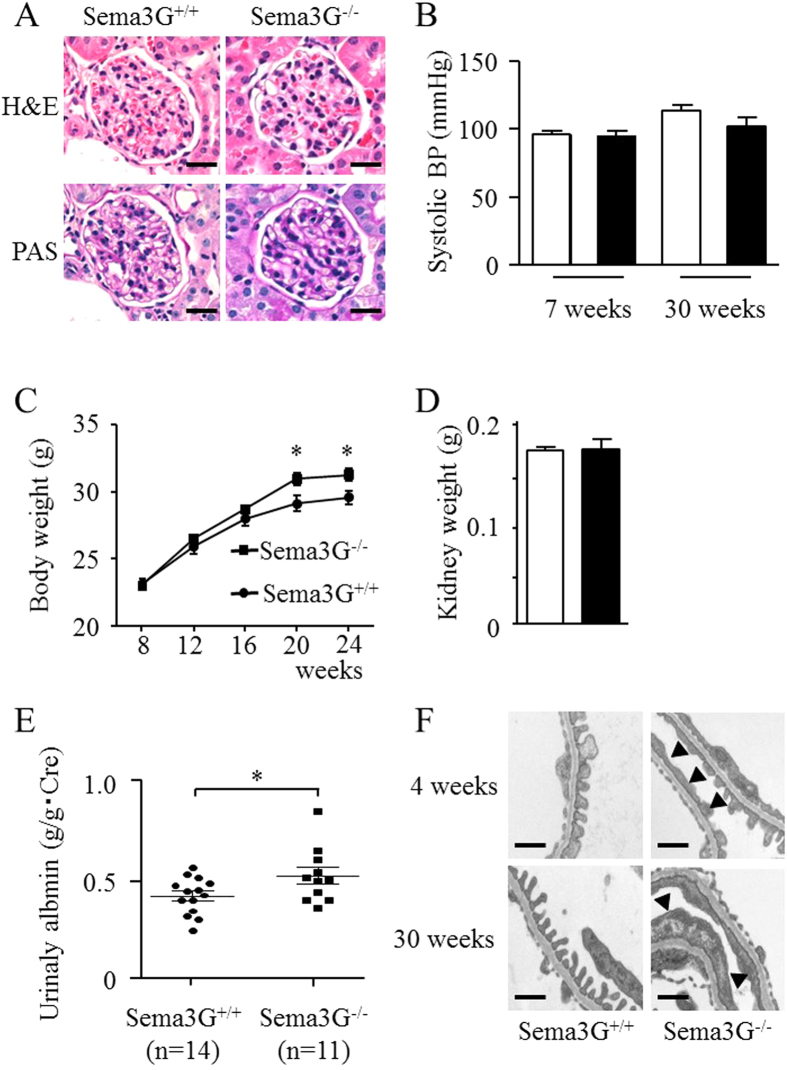
Phenotypic analyses of Sema3G mutant animals. Sema3G knockout mice were created by ordinary homologous recombination. (**A**) The histological examination of 12-week-old mouse kidney was done by light microscopy. (**B**) Blood pressure (n = 6 mice in each), (**C**) Body weights (14 mice for Sema3G knockout mice and 13 mice for littermate controls), (**D**) Kidney weights (12-week-old mice, n = 4 mice in each) were evaluated. (**E**) The amounts of albuminuria and urinary creatinine in 10-week-old mice were evaluated by ELISA. (**F**) The histological examinations of 4- and 30-week-old mouse kidney were done under the electron microscope (n = 2 mice in each). Arrowheads indicate the foot process effacement of podocytes in the Sema3G knockout mice. Representative pictures were shown. Scale bars: 20μm (A), 500nm (F). *P < 0.05.

We generated Sema3G mutant mice by the use of VelociGene technology^11^ to investigate the functions of Sema3G *in vivo*. The Sema3G gene comprises 16 exons. Bacterial artificial chromosome-based targeting vectors were used to specifically replace the coding region from exon 2 to exon 16 with beta-galactosidase-encoding sequences (LacZ) and a loxP-flanked neomycin selection cassette. Chimeric mice derived from two independent ES clones were bred to C57BL/6 males to produce F1 offspring. Next, the Sema3G^LacZ/+^ mice were intercrossed to produce homozygous mutant offspring. The absence of Sema3G mRNA in the mutant mice was confirmed by reverse transcription polymerase chain reaction (RT-PCR) with cDNA generated from glomerular RNA of 5-week-old animals (unpublished observation). Mice homozygous for the inactivated Sema3G allele were viable and fertile and did not display any obvious developmental or behavioral defects. Light microscopic analysis revealed that the Sema3G null mice had no observable glomerular developmental defects (Fig. 3A).

**Figure 4:**
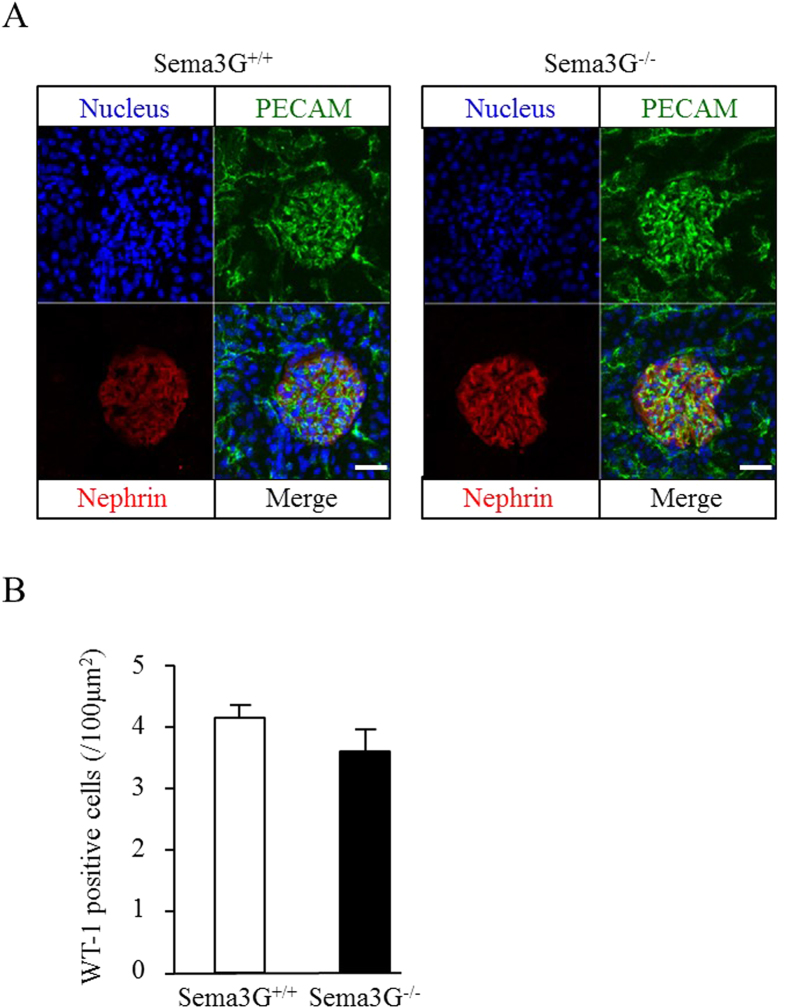
Structure of Sema3G knockout glomeruli were evaluated by immunofluorescence. (**A**) Kidneys were dissected from 10-week-old mice subjected for immunofluorescence. Anti-PECAM antibody was used to evaluate glomerular capillary structure and anti-nephrin antibody was used for podocytes. At least three animals were used for each experiment and representative pictures were shown. (**B**) The numbers of podocytes per glomeruli were evaluated by the WT-1 immunostaining. Twenty glomeruli were taken from one mouse at random. Three 10-week-old Sema3G knockout mice and littermate controls each were used.

Since Sema3G is expressed in vascular-enriched tissues, we measured the blood pressure of the adult mice and did not find any difference between the Sema3G mutant animals and wild-type controls (Fig. 3B). There were no differences in the body weights between Sema3G mutant animals and litter mate controls until 16 weeks after birth, however Sema3G mutant animals became slightly heavier than the litter mate controls 20 weeks after birth (Fig. 3C). There were no differences in the kidney weights between Sema3G mutant animals and litter mate controls (Fig. 3D). We evaluated urinary albumin excretion by ELISA in 10-week-old animals and found that Sema3G knockout animals had slight albuminuria, but significantly more than the controls (Fig. 3E). Electron microscopic study revealed localized foot process effacement of the podocytes that was worse in the knockouts as the mice aged (Fig. 3F). However, the difference in the albuminuria between the knockouts and wild-types did not change with age (unpublished observation). We also examined and found no significant differences in the glomerular capillary structure with immune-staining with anti-PECAM antibody and the podocyte structure with anti-nephrin antibody (Fig. 4A). The number of podocytes within the glomeruli was also counted by Wilms tumor-1 (WT-1) immune staining and no significant difference was found (Fig. 4B). Because it was reported that semaphorins play a role in neuron guidance, we stained peripheral neurons in the whole mount with anti-Tuj-1 antibody and observed no significant differences (Supplemental Figure 3).

### Sema3G knockout mice produce more albuminuria during acute kidney inflammation and under diabetic conditions

**Figure 5:**
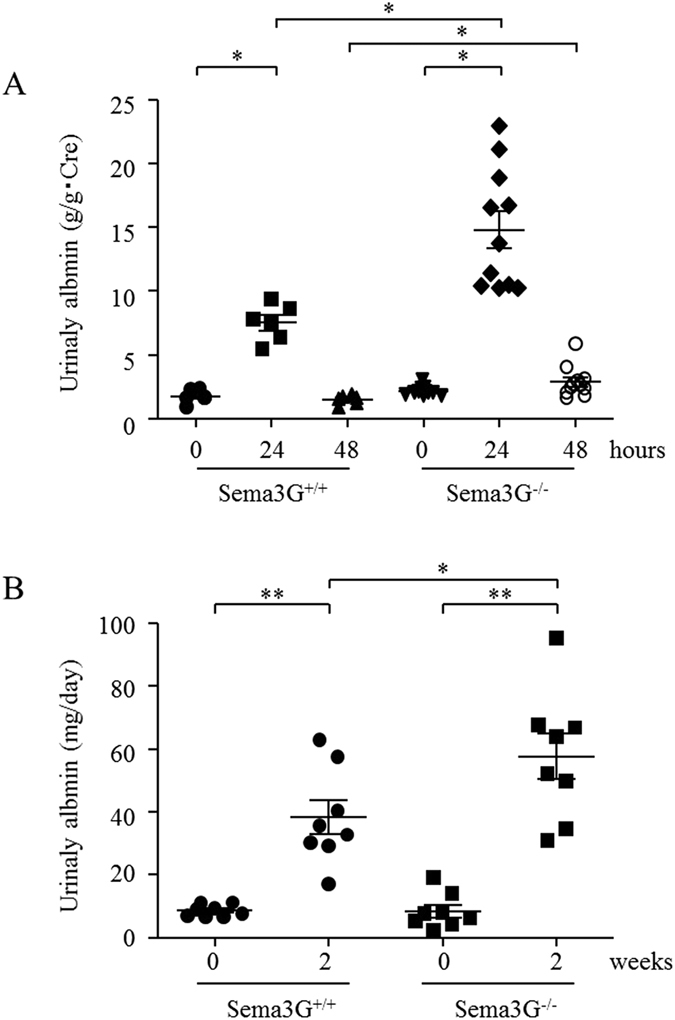
Sema3G may protect glomeruli from inflammatory and diabetic injuries. When inflammation was induced by LPS injection (**A**) or diabetes was induced by streptozotocin injection (**B**), the Sema3G null mice produced higher degrees of albuminuria compared with the wild-type controls. *P < 0.05,** P < 0.01. (A) 8-week-old mice, 6 Sema3G+/+, and 11 Sema3G−/− were used. (B) 8-week-old mice, 8 Sema3G+/+, and 8 Sema3G−/− were used.

Because we could observe only subtle phenotypes in Sema3G knockout mice in physiological conditions, we applied two disease models in order to investigate the roles of Sema3G under disease conditions. First, we performed intraperitoneal injection of lipopolysaccharide (LPS) into mice as a model for acute inflammation. This model reportedly induces inflammatory cytokines, podocyte injuries, and transient albuminuria^12^. We found that Sema3G mutant animals produced more albuminuria and took longer time to normalize (Fig. 5A). Next, we induced diabetes by injecting mice with STZ and found that diabetic Sema3G mice transiently produced more albuminuria than the wild-type controls 2 weeks after the STZ injection (Fig. 5B). However, they returned to the level of the wild-type controls 4 weeks after the STZ injection (unpublished observation). These results indicated that Sema3G acted as an anti-inflammatory and anti-diabetic within the glomerulus.

### Sema3G knock out podocyte expressed inflammatory cytokines

Because the difference of albuminuria between Sema3G mutant animals and wild type controls was observed more in the LPS-induced acute inflammation models than the diabetic models, we opted to analyze the anti-inflammatory mechanisms rather than the anti-diabetic mechanisms of Sema3G *in vitro*. We established Sema3G knockout podocytes and wild-type podocytes and analyzed the expression of inflammatory cytokines. The establishment of cultured podocytes was evaluated by the amplification of podocyte-specific markers by RT-PCR and immunostaining with anti-WT-1 and anti-synaptopodin specific antibodies (Supplemental Figure 4A,B).

**Figure 6:**
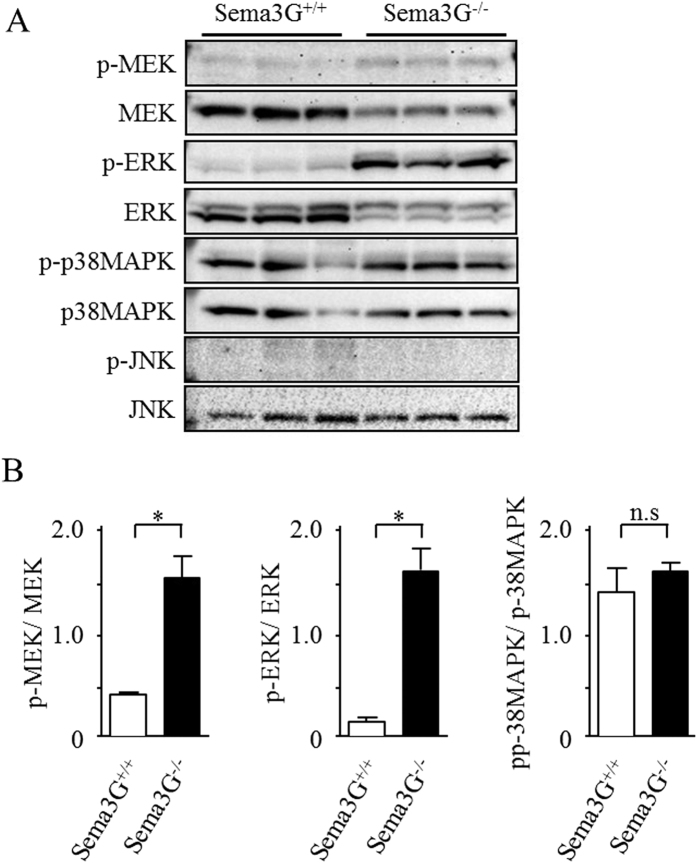
Effects of Sema3G deficiency in cultured podocytes on intracellular signaling pathways. (**A**,**B**) Differentiated podocytes were lysed for immunoblotting and assessed for MEK, ERK, p38MAPK and JNK phosphorylation. *P < *0.05, n.s.: not significant*.

**Figure 7:**
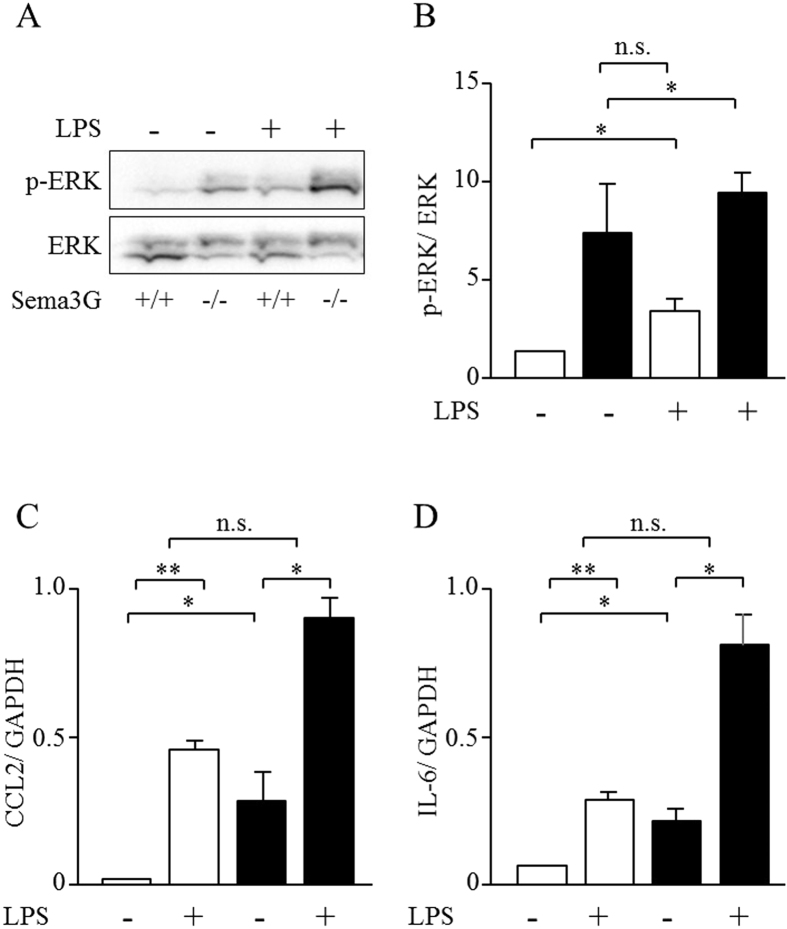
Effects of Sema3G deficiency on ERK activation and the expression of inflammatory cytokines, induced in the presence of LPS in cultured podocytes. Podocytes were stimulated with 0.1 μg/mL LPS for 30 min. Cells were lysed for immunoblotting and assessed for ERK1/2 phosphorylation (**A**,**B**), or the expression of CCL2 and IL-6 by real-time PCR (**C**,**D**). *Open columns: Sema3G*^+/+^* podocytes, Closed columns: Sema3G*^*−/−*^podocytes. *P < 0.05, **P < 0.01. Experiments were repeated at least three times and bars represent mean ± SEM. n.s.: not significant.

We performed transcriptional analysis of Sema3G knockout podocytes and wild-type controls using cDNA microarray and found that inflammatory cytokines, such as chemokine (C-C motif) ligand 2 (CCL2) and interleukin 6 (IL-6), were upregulated in Sema3G knockout podocytes compared with wild-type controls (Supplemental Table 1). Subsequently, several intracellular signaling pathways were analyzed, and we found that the phosphorylation of mitogen-activated protein kinase kinase (MEK) and extracellular signal-regulated kinase (ERK), but not p38 mitogen-activated protein kinase (p38 MAPK) and c-Jun N-terminal kinase (JNK), was increased, as shown in Fig. 6. Furthermore, we analyzed the effects of LPS on the expression of inflammatory cytokines in both Sema3G knockout podocytes and wild-type control podocytes. As shown in Fig. 7, not only the phosphorylation of ERK but also the expressions of CCL2 and IL-6 were significantly increased in Sema3G knockout podocytes compared with wild-type podocytes, thus confirming the results of the microarray. However, in the presence of LPS, the expressions of those cytokines and the phosphorylation of ERK increased to a similar level in Sema3G knockout podocytes and wild-type podocytes (Fig. 7). These results indicated that Sema3G secreted from podocytes inhibited basal inflammatory cytokine expression in a podocyte autocrine and/or paracrine manner. However, the endogenous levels of Sema3G in wild-type podocytes might not be enough to inhibit LPS-induced expression of inflammatory cytokines.

### Sema3G suppresses inflammation through inhibition of TLR-4 signaling

**Figure 8:**
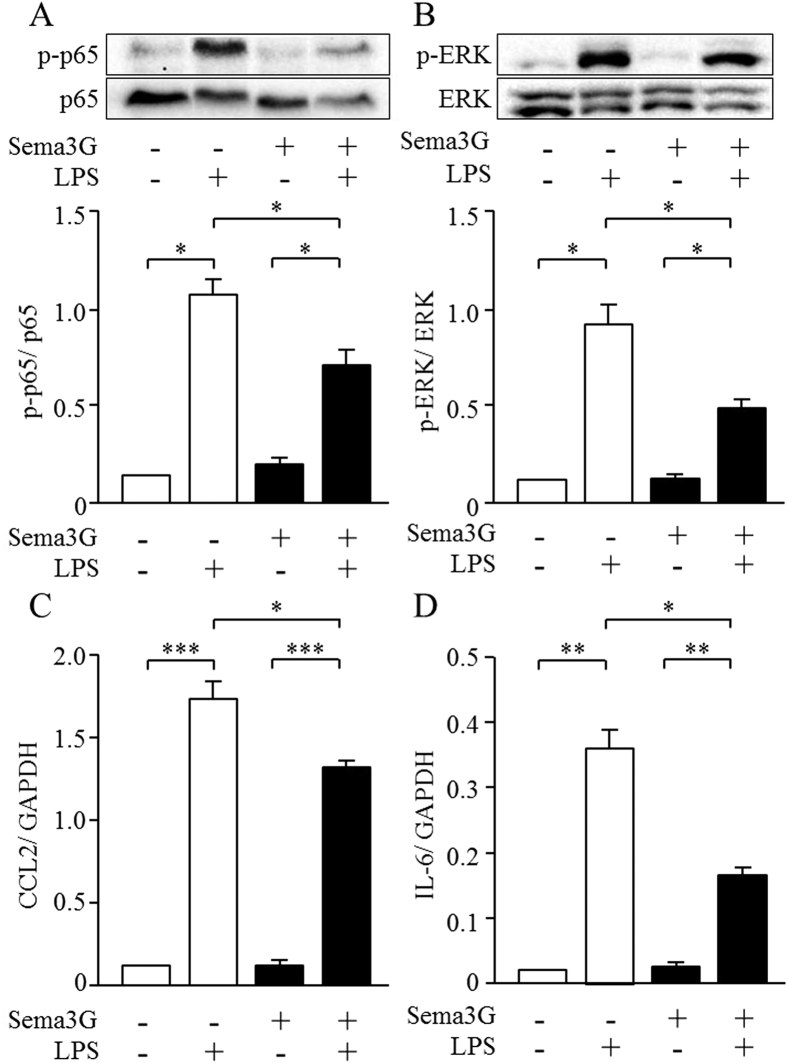
Sema3G inhibited the phosphorylation of p65, ERK, and the expression of inflammatory cytokines, which were activated by LPS. (**A**,**B**) Podocytes were pretreated with 0.05 μg/mL Sema3G protein for 1 h and then stimulated with 0.1 μg /ml of LPS for 30 min. Phosphorylation of p65 and ERK was evaluated by Western blotting. (**C**,**D**) Podocytes were pretreated with 0.05 μg/mL Sema3G protein for 1 h and then stimulated with 0.1 μg /ml of LPS for 3 hrs. The expression of CCL2 and IL-6 was evaluated by real-time PCR. *P < 0.05, **P < 0.01, ***P < 0.001. Experiments were repeated at least three times and bars represent mean ± SEM.

Next, we produced Sema3G recombinant protein (r-Sema3G) and examined its anti-inflammatory effects. The presence of r-Sema3G significantly attenuated the LPS-induced increased expression of inflammatory cytokines and phosphorylation of ERK and p65, a component of NF-κB signaling (Fig. 8) but not the phosphorylation of c-jun N-terminal kinase (JNK) and p38MAPK (Supplemental Figure 5). These results suggested that Sema3G attenuated LPS-induced inflammatory cytokines through the inhibition of ERK and NF-κB signaling.

## Discussion

We have identified a new podocyte-expressed gene, Sema3G. It is a secreted protein.

The glomerular function was worse in mice lacking Sema3G when we induced acute inflammatory kidney injury and diabetes. Sema3G knockout podocytes produced more inflammatory cytokines as compared to the control. Sema3G acted as an anti-inflammatory which was induced by LPS.

The number of patients who suffer from kidney disease has been increasing worldwide. The most common cause of kidney disease is DN; however, its precise pathophysiology remains unclear. It has been reported that strict control of blood glucose, blood pressure, and blood lipid levels delay the development and/or progression of DN. Furthermore, some of the anti-hypertensive drugs, such as angiotensin II type 1 receptor blockers and angiotensin converting enzyme inhibitors, might delay the development of kidney diseases^13^. However, none of these treatments of kidney disease are specific to the glomerulus.

It has recently been reported that inflammation is a key factor in kidney disease and a wide variety of other diseases such as atherosclerosis^14^, obesity^15^, and cancer^16^. Among the inflammatory signals, TLR4 signals, which are an innate immune response, reportedly affect the development of various kidney diseases, including DN^17^. Pathogen-associated molecular patterns classically activate TLR4, but it is also activated by high levels of glucose^18^, fatty acids^19^ and fibrinogen^20^. Diabetic complications are attenuated by blockade of TLR signaling either pharmacologically, with the potent immune-modulator (S, R)-3-phenyl-4, 5-dihydro-5-isoxasole ascetic acid (VGX-1027)^21^, or by gene targeting, such as that observed in TLR4 knockout mice^22^. Therefore, TLR-4 signaling becomes a very important target for the prevention and treatment of kidney disease. However, systemic inhibition of the innate immune system has shown a tendency to increase the risks of side effects, such as bacterial infections.

Because the glomerulus is a culprit for most kidney disease, it is important to know which cell types within the glomerulus express TLR. TLR-4 has been reported to localize to glomerular podocytes in a wide variety of kidney diseases^17^,^23^. Podocytes play pivotal roles in the filtration of blood through the slit diaphragm between the podocyte foot processes^24^. Podocytes also secrete a number of growth factors that are important in maintaining the glomerular capillary tuft. Thus, the dysregulation of podocyte-secreted factors, which might be called as “podocytokines”, contributes to the development of glomerular disease. It follows that it is very important to protect podocytes from inflammatory injuries.

Semaphorins bind to plexins and regulate key cellular functions, including cell-cell interaction, and cellular differentiation, proliferation, and migration^8^. Therefore, semaphorin and plexin signals have become therapeutic targets for cancer^25^,^26^, bone disease^27^, microvascular disease^28^, and inflammatory disease^29^. It has been reported that semaphorin signals are linked with both the adaptive and innate immune response. For instance, it has been reported that the semaphorin 3A-plexin A4 signal enhanced TLR4 signaling^30^. It has also been reported that semaphorin plays a pivotal role in kidney disease. Among the semaphorins, Sema3A has been the best characterized semaphorin concerning DN. It has been reported that urinary Sema3A correlates with diabetic proteinuria and mediates DN and the associated inflammation in mice^31^,^32^. Therefore, an increased expression of Sema3A worsens DN through the increased expression of inflammatory cytokines. Further, it has been reported that not only Sema3E^33^ but also Sema7A together with its receptor, Plexin C1^34^,^35^ promote inflammation.

It has been reported that the expression of Sema3G was decreased in diabetic kidney diseases^36^. Our results indicated that lack of Sema3G in podocytes induced the expression of low levels of inflammatory cytokines. These ‘chronic low grade inflammation’ within glomeruli might cause podocyte structural changes which potentially lead to produce albuminuria which were seen in Sema3G knock out animals. It would be interesting to speculate that a balance between the expression of the other semaphorins including Sema3A, which is pro-inflammatory, and Sema3G, which is anti-inflammatory, is important in the development of kidney injuries. Indeed, not only the lack of Sema3G but also the induction of inflammation by LPS widely affected the expressions of other semaphorins and its receptor such as neuropilins and plexins in podocytes as shown in Supplemental Table 1.

The mechanisms by which Sema3G inhibits LPS-induced TLR4 have not yet been completely elucidated. It has been confirmed that Sema3G binds with neuropilin 1 and/or 2^37^. And all the cell types within glomerulus expressed neuropilin 1 but not neuropilin 2. There is almost no intracellular domain in neuropilin, meaning that neuropilin itself does not work as a signaling receptor but rather works as a co-receptor with other receptors such as plexins. It has been reported that neuropilins are required for the activation of class A plexins. Therefore, it has been assumed that Sema3G binds with neuropilin and subsequently activates plexins, which results in intracellular signaling. The cytoplasmic regions of plexins contain two regions that are homologous with small guanine triphosphate hydrolase-activating protein. The stimulation of plexin A4 with Sema3A has been reported to subsequently activate Rac1, leading to the activation of NF-kB and JNK. This plexinA4-Sema3A signaling engaged with enhancement of TLR signaling in monocytes^30^. Therefore, Sema3G might work as an antagonist to plexinA4 signaling through the inhibition of receptor binding with other ligands or the direct inhibition of the intracellular signaling cascade. Further analyses are necessary to prove these hypotheses.

We believe that Sema3G might be a drug target because it attenuates inflammatory cytokines which were induced by LPS. The gene regulations of Sema3G have not yet been completed. It has been reported that peroxisome proliferator-activated receptor-gamma (PPAR-γ) increased the expression of Sema3G in cultured endothelial cells^38^. There are several reports that the PPAR-γ agonist has renoprotective effects^39^,^40^. The renoprotective effects of PPAR-γ might partially depend on the effects of Sema3G.

There are several limitations to this study. We showed that a lack of Sema3G in mice worsened kidney function but we did not show the protective effects of Sema3G when overexpressed or administered to mice. Since all the cell types within glomerulus expressed neuropilin 1, Sema3G might also affect the functions of endothelial cells and mesangial cells in paracrine fashion. However, these possibilities have not analyzed, yet. We have not yet demonstrated how Sema3G attenuates inflammatory cytokines.

In conclusion, we have identified a novel podocyte-specific gene, Sema3G. This gene inhibits LPS-induced inflammation. Our results suggest that podocyte-specific therapy is a viable option to inhibit inflammatory kidney disease, including DN, in the near future. Further research on Sema3G is needed to confirm our findings and hypotheses.

## Materials and Methods

### Reagents

Lipopolysaccharide (LPS) was obtained from Sigma Aldrich (#L4524). Streptozotocin was also from Sigma Aldrich (#S0130).

### *In situ* hybridization

Paraffin-embedded block and section (6 μm) of mouse embryonic day 18.5 (E18.5) for *in situ* hybridization (ISH) was obtained from Genostaff Co., Ltd. See the online supplemental materials for details.

### Northern blotting

Total RNA was isolated using the RNeasy mini kit (Qiagen Inc., Valencia, CA), according to the manufacturer’s instruction. Northern blot analysis was performed, as described previously^41^, using ^32^P-labeled Sema3G cDNA and glyceraldehyde-3-phosphate dehydrogenase (GAPDH) cDNA probes. FirstChoice Mouse Blot I was obtained from Ambion.

### Isolation of glomeruli

The glomeruli were isolated by Dynabeads (Invitrogen, Norway) perfusion technique, as previously described^41^.

### Sema3G stable expression

Full coding sequence of human Sema3G (Genecopoeia; EX-T0068-M02) was subcloned into a retrovirus vector pQCXIN (Clontech), according to the manufacturer’s instructions. PT-67 cells were transiently transfected with pQCXIN Sema3G construct. Cell-free viral supernatants were harvested at 24 and 48 h, mixed with one volume of complete media, in the presence of 8 μg/mL polybrene (Sigma, USA), and used for transfection into Cos7. Sema3G-stable-expression-cells were selected by 500 μg/ml of G418 (Promega).

### Western blotting

Cells were lysed in an SDS sample buffer containing 0.5 M Tris–HCl, 10% SDS, glycerol, bromophenol blue, and 3% 2-mercaptoethanol. They were boiled at 95 °C for 5 min, and then the protein was fractionated on 7.5–12.5% SDS-polyacrylamide gel electrophoresis (PAGE) (e-PAGEL, ATTO Corporation, Japan). The protein was transferred to PVDF membranes (Immobilon-P Transfer Membrane): the membranes were blocked for 1 h at room temperature to block the non-specific binding of the protein and incubated with primary antibodies at 4 °C overnight. The primary antibodies were as follows: anti-sema3G antibody (Sigma Aldrich, HPA001761, 1:1000 dilution), anti-phospho-p65 antibody (Santa Cruz, sc-101749,1:1000 dilution), anti-p65 antibody (Santa Cruz, sc-372, 1:1000 dilution), anti-phospho-ERK antibody (Cell Signaling, #9106, 1:1000 dilution), and anti-ERK antibody (Cell Signaling, #9102, 1:1000 dilution). The blots were then washed and incubated with second antibodies, peroxidase-conjugated anti-rabbit immunoglobulins (1:2500 dilution, GE healthcare), or goat anti-mouse IgG-HRP (1:2500 dilution, Santa Cruz: sc-2055) at room temperature for 1 h. After washing several times, the antibody binding sites were visualized using an ECL Western blotting detection system (GE healthcare: RPN2106). The blots were quantified using a ChemiDoc MP ImageLab PC system (BIO-RAD).

### Physiological studies in mice

Blood pressure was measured in conscious male and female mice by tail cuff plethysmography, according to the manufacturer’s specifications.

### Transmission Electron Microscopy

Mice were fixed in 4% paraformaldehyde in PBS solution by perfusion fixation. Tissue samples were collected and fixed in phosphate-buffered 2.5% glutaraldehyde (pH 7.4), post osmicated, and dehydrated with graded alcohol. After immersion in propylene oxide, the specimens were embedded in Epon 812. Ultrathin sections were prepared and collected on electron microscopic grids and examined with a transmission electron microscope (JEM-1011; JEOL).

### Immunohistochemistry

The kidney tissues were dissected from the mouse, fixed in O.C.T. compound, and stored at −80°C until use. Several 4–8 μm frozen sections were prepared and fixed with 4% paraformaldehyde or ice-cold methanol and then blocked with blocking buffer, containing 2% bovine serum albumin (BSA), and 0.05% Tween-20 in PBS. After washing with Tween in PBS (PBST; 0.1% Tween-20 in PBS), the slides were co-incubated with nephrin antibody (1:500 dilution), PECAM antibody (BD Pharmingen #550274, 1:200 dilution), WT-1 antibody (Santa Cruz, sc-129, 1:50 dilution), Neuropilin-1 antibody (kindly provided by Dr. Fumikazu Sudo, National Institute of Neuroscience, Japan, 1:200 dilution) and Hoechst for nuclear staining. The slides were imaged by the Axio Observer D1 (ZEISS) or with a confocal laser-scanning microscope (Leica LSM5 PASCAL). The images were processed using Adobe Photoshop.

### Animals

Male C57BL/6J mice were obtained from Clea Japan. Sema3G mutant animals were created as previously described^37^. Artificial lighting was maintained on a 12:12-h light–dark cycle. Approval was obtained from the Chiba University Ethics Committee, and all experiments were performed in accordance with specified guidelines for the care and use of laboratory animals.

### Genotyping of mutant mice

PCR amplification of genomic DNA extracted from tail biopsies was used to genotype the Sema3G^LacZ/LacZ^ mice as previously described^37^.

### Induction of albuminuria by the injection of LPS

Eight-week-old, male Sema3G-deficient mice and wild-type mice were injected with 13 mg/kg of LPS in 200 μl of PBS or 200 μl of control PBS by intraperitoneal administration as described previously^12^. The spot urine samples were collected before and 24 h after injection.

### Induction of diabetes by the injection of streptozotocin

Eight-week-old, male Sema3G-deficient mice and wild-type mice were injected with 200 mg/kg of streptozotocin by intraperitoneal administration. STZ was dissolved in an ice-cold citrate buffer at pH 4.6 and immediately injected. Pooled urine samples for 24 h were collected before injection and weekly thereafter.

### Measurement of albuminuria

The amount of albumin excreted was examined by Mouse Albumin ELISA KIT (Shibayagi #AKRAL-121), according to the manufacturer’s instructions. Spot urine samples were corrected by creatinine, using The Creatinine Companion (Exocell #1012), according to the manufacturer’s instructions. Pooled urine samples were corrected by the amount of urine samples for 24 h.

### Cultured human podocytes and Sema3G-deficienct murine podocytes

Sema3G knockout mice were mated with SV40 large T-antigen transgenic mice [Immortomouse: CBA; B10-Tg (H2Kb-tsA58) 6Kio/Crl], purchased from Charles River. Heterozygous F1 mice were crossed to obtain Sema3G^LacZ/LacZ^/SV40 large T+ mice. Glomeruli were isolated from these mice, as described previously^41^, and Sema3G deficiency podocytes were cultured from isolated glomeruli. Wild-type podocytes were generated from littermate wild-type controls. Podocytes were then differentiated under permissive conditions for 7 to 10 days. After differentiation, the culture media was replaced by RPMI1640 containing 0.1% BSA for 24 h, and then used for the experiments. Human podocytes were kindly provided by Dr. MOIN A. SALEEM in University of Bristol, Southmead Hospital and were differentiated according to the protocol described earlier^42^. After differentiation, cells were incubated for 48 h in a medium without serum, and the medium was used as a conditioned medium.

### Microarray analysis

Microarray analyses were done by TAKARA Bio Inc. using Agilent Expression Array (SurePrint G3 Mouse GE 8 × 60 K Microarray).

### Sema3G recombinant protein

Full-length Sema3G recombinant protein (human) was produced by FreeStyle293-F (Invitrogen) overexpression, which was tagged by flag tag in the N terminal. Three μmol/L of Furin inhibitor (Dec-RVKR-CMK: Biomol) was added in media 0, 24, 48, and 72 h after overexpression. The purified protein was sterilized by filtration.

### Real-time Polymerase Chain Reaction (Real-time PCR)

Real-time PCR was carried out as previously described^43^. The primers used were as follows: Mouse-Sema3G Forward, GAA GCC GAG ATG CCC TTT AC and Reverse, GTC TTT TCC CTT GCG GAC AC; Mouse-GAPDH Forward, AAC TTT GGC ATT GTG GAA GG and Reverse, GGA TGC AGG GAT GAT GTT CT; Mouse- IL-6 Forward, CAACGATGATGCACTTGC and Reverse, GTACTCCAGGTAGCTATG; and Mouse-CCL2 Forward CCTGCTGTTCAC AGTTGCC and Reverse, ATTGGGATCATCTTGCTGGT. The gene expression was calculated by the comparative ΔΔCt method, using 7500 Fast System SDS software (Applied Biosystems).

### Statistical analysis

Student’s unpaired t-test was performed to compare differences between the groups, and P < 0.05 was defined as statistically significant.

## Additional Information

**How to cite this article**: Ishibashi, R. *et al*. A novel podocyte gene, semaphorin 3G, protects glomerular podocyte from lipopolysaccharide-induced inflammation. *Sci. Rep.* **6**, 25955; doi: 10.1038/srep25955 (2016).

## Supplementary Material

Supplementary Information
