# Supplementary material for: A novel podocyte gene, semaphorin 3G, protects glomerular podocyte from lipopolysaccharide-induced inflammation
Source: Sci Rep. 2016 May 16;6:25955. doi: 10.1038/srep25955 (PMC4867620; doi:10.1038/srep25955)
Supplement: Supplementary Information [file srep25955-s1.pdf]

On Line Supplement

A novel podocyte gene, Semaphorin 3G, protects glomerular podocyte from lipopolysaccharide-induced inflammation

Ryoichi Ishibashi, Minoru Takemoto, Yoshihiro Akimoto, Takahiro Ishikawa, Peng He, Yoshiro Maezawa, Kenichi Sakamoto, Yuya Tsurutani, Shintaro Ide, Kana Ide, Harukiyo Kawamura, Kazuki Kobayashi, Hirotake Tokuyama, Karl Tryggvason, Christer Betsholtz and KoutaroYokote

Materials and Methods

In situ hybridization

For ISH, mouse E18.5 sections were de-waxed with xylene, and rehydrated through an ethanol series and phosphate buffer solution (PBS). The sections were fixed with 4% paraformaldehyde in PBS for 15 min and then washed with PBS. The sections were treated with 8 μg/ml Proteinase K in PBS for 30 min at 37 °C, washed with PBS, re-fixed with 4% paraformaldehyde in PBS, washed again with PBS, and placed in 0.2 N HCl for 10 min. After washing with PBS, the sections were acetylated by incubation in 0.1 M tri-ethanolamine–HCl, pH 8.0 and 0.25% acetic anhydride, for 10 min. After washing with PBS, the sections were dehydrated through a series of ethanols. Hybridization was performed with probes at concentrations of 300 ng/ml in the Probe Diluent-1 (Genostaff), at 60 °C for 16 hr. After hybridization, the sections were washed in 5×HybriWash (Genostaff), equal to 5×SSC, at 60 °C for 20 min and then in 50% formamide,

2×HybriWash at 60 °C for 20 min, followed by RNase treatment in 50 μg/ml RNase A in 10 mM Tris–HCl, pH 8.0, 1 M NaCl, and 1 mM EDTA for 30 min at 37 °C. Then the sections were washed two times with 2×HybriWash at 60 °C for 20 min, two times with 0.2×HybriWash at 60 °C for 20 min, and one time with TBST (0.1% Tween20 in TBS). After treatment with 1×G-Block (Genostaff) for 15 min at RT, the sections were incubated with anti-DIG AP conjugate (Roche) diluted 1:2000 with ×50G-Block (Genostaff) in TBST for 1 h at RT. The sections were washed two times with TBST and then incubated in 100 mM NaCl, 50 mM MgCl2, 0.1% Tween20, and 100 mM Tris-HCl, pH 9.5. Coloring reactions were performed with NBT/BCIP solution (Sigma) overnight and then washed with PBS. The sections were counterstained with Kernechtrot stain solution (Mutoh), dehydrated, and mounted with Malinol (Muto). The sequences used are shown in supplemental Table 2.

ENSEMBL ID	GENE SYMBOL	1. Sema3G$^{-/-}$/Sema3G$^{+/+}$	2. LPS+/LPS-
ENSMUST00000120243	Ccl2	2.1	5.1
ENSMUST00000026845	Il6	2.8	10.7
ENSMUST00000026917	Nrp1	-1.2	-1.6
ENSMUST00000027112	Nrp2	1	1.6
ENSMUST00000163139	PlxnA1	-1.1	-0.7
ENSMUST00000125381	PlxnA2	-1.5	-1.7
ENSMUST00000154062	PlxnaA3	-2.2	1
ENSMUST00000115096	PlxnA4	-1	-1.1
ENSMUST00000072093	PlxnB1	-1.5	-2
ENSMUST00000060808	PlxnB2	-1	-1
ENSMUST00000149478	PlxnB3	-2.9	-1.8
ENSMUST00000099337	PlxnC1	-5.5	-14
ENSMUST00000015511	PlxnD1	-1.3	1.1
ENSMUST00000095012	Sema3A	-1.1	-1.6
ENSMUST00000102530	Sema3B	-1.9	-1.5
ENSMUST00000115271	Sema3C	-1.7	1.5
ENSMUST00000030868	Sema3D	-6.7	-5.3
ENSMUST00000073957	Sema3E	1.1	-0.6
ENSMUST00000080560	Sema3G	-1	-2.3
ENSMUST00000090180	Sema3G	-33.6	-3
ENSMUST00000165898	Sema4A	1.2	2
ENSMUST00000114990	Sema4C	1.1	2.2
ENSMUST00000021900	Sema4D	1.1	-1.1
ENSMUST00000000641	Sema4E	-1.4	-2.5
ENSMUST00000179305	Sema4G	-1.4	-4.8
ENSMUST00000067458	Sema5A	1.5	-1.9
ENSMUST00000050625	Sema5B	1.1	1.1
ENSMUST00000076043	Sema6A	1.2	-1.1
ENSMUST00000043059	Sema7A	2.2	2.4

Supplemental Table 1. Microarray analyses.

1. RNA was isolated from Sema3G$^{+/+}$ and Sema3G$^{-/-}$podocyte.

2. RNA was isolated from with or without 0.1 μg/ml LPS stimulated podocytes.

Fold changes were shown.

Probe1	TCGAGGTGTATGCGCTGTTCAGCACTGTCAGTGCTGTGTTCCAGG GCTTTGCTGTCTGTGTGTACCACATGGTAGACATCTGGGAGGTCT TCAATGGGCCCTTTGCCCACCGAGATGGCCCTCAGCATCAGTGG GGACCCTATGGGGGCAAGGTGCCCTTCCCTCGCCCCGGTGTGTG TCCTAGCAAGATGACCGCACAGCCAGGCCGACCCTTTGGCAGCA CCAAGGACTACCCAGACGAGGTGTTGCAGTTTGTCCGAGACCAC CCACTCATGTTCCAGCCTGTGAGGCCTCGGCGTGGCCGCCCTGT CCTGGTCAAAACTCACTTGGCTCAGCGACTGCGCCAGATTGTGG TGGATCGAGTGGAGGCTGAGGATGGGACCTATGATGTCATCTTCC TAGGGACTGATTCGGGTTCTGTGCTCAAAGTCATTGCCCTCCAGG GTGGTGGCTTGACTGAACCTGAAGAGGTGGTTTTGGAGGAGCTC CAGGTGTTTAAGGTGCCAACGCCCATCACTGAGATGGAGATCTCT GTCAAAAGGCAAACGCTGTATGTGGGCT
Probe2	CAGGGGATAAGGGGACTGACCAGGTGAAGACAGATGAGAGAGT TGTGCAGACGGCCCAAGGGCTGCTGTTCCGAAGGCTCAGCCGCC ATGACGCAGGAAACTACACTTGCACCACTCTGGAACATGGCTTC TCCCAGACCGTGGTCCGTTTTGCCCTGGAGGTGATTGCAGCTGT GCAACTGGACAGCCTGTTCCTTCGGGAGTCAAGGCTAGAGGAGC CCTCAGCCTGGGGAAGCCTGGCCTCTGCCTCCCCCAAGACTTGG TATAAGGACATCCTTCAGCTCACGGGCTTCGCCAACCTGCCCCGT GTGGATGAGTACTGTGAACGTGTATGGTGCAGGGGTGTCGGGGA GCGCTCAGGCTCCTTCCGCGGAAAGGGAAAGCAAGCGAAGGGC AAGAGCTGGGCAGGGCTGGAACTGGGCAAGAAGATGAAGAGCA GGGTGCTGGCTGAGCACAATCGGACACCCCGGGAGGTAGAAGC CACATAGAAGATGACTGAGGAGCAGGTGGTCGGGCTGGGCTGG GGGACTCAACGGTATCTCTCTCCCACCCAGCTAGGAAAGAGGAG

	TCCAGAACTCCTGATTGATTACCTCTTAGAGGAAATCTCCACCAC CTACAGGAAAGACTAGGGGTTGGTGGGAGGAGCCTTGCACCTCA GCCTACCCCTGCCTTTCTCCGAGTTCTCAGTCCCGGGCTGGAACT GGCACCTCCAGACCACTTGT
Probe3	ATTTCCGGTTGTGCCCAGAGGCAAGGCGGGTGGTTCTTTCCCAG CTTGCTGAACAATGGCCATTCTGAGTGACCCTCTGAGTGGGTGTA TGGGTGGCTCTAAGGGAGTATATATTGTCAGTCCACCAGAGGAGG GGAAAGTAGATTCCTAAGGTAGAACCTGATAAGAAACACTTGCC CCACAGAGGTGTGAGAGAAGGTGGAGGCGCTGAGGAGGTGGGG TTGTGCTGTTTATCTGTCTCCATGCTTTGGAATCTAGATGATTCCC TGCCTCAGCTCCCTCCCATCTCCTACTCACAGAGGCAGGAACTGC TGGGACCTGGAGGTTGCCTGGGCCTGGCCCTTGCCCCCTTTTTAA ACTTGGAAGAGTTGTCTCACAATATGATACCCTTTCCCAGGTCCC ACATTGTCAGGCCAGGCTTGCTGGGGTGCTCACACCTCACCATC AGAGTGTCTTCCTGGGACCAGGGGAAGGTTCTGTGGACCAGAA AGAGGAAGTGGGGAGCTGGAGGGTAGGAGTGCCTCGAAGGATG GGCTACCTTTCCGAGGGTGAGGCGGGCATGCTCAGGCCGGTGAG GTTAGCAACGGACACTCTGAAAACCAGCAGCCAGTGGTGAGGAT CGGCCTCCTGGGCTTTGTCTAGGGCCACGAGGAACTAGAGAGAG GTG

Supplemental Table 2.

Three different sequences were used for ISH. Sequences for anti-sense probes used were as follows.

Supplemental Figure 1.

In situ hybridization on E18.5 mouse embryo for Sema3G sense probes

The sense probes for Sema3G gave no signals.

(A) Bar indicated 1mm, (B) Bar indicated 400μm, (C) Bar indicated 200 μm,

(D) Bar indicated 50μm.

Supplemental Figure 2.

There were no distinct differences in neuronal patterning in Sema3G knockout mice

Whole mount Tuj-1 and anti-neuron specific beta III isoform of tubulin staining was performed in post-natal, day 0 kidneys. Whole mount immunohistochemistry were done as previously described[1].

(A) Bar indicated 500μm, (B) Bar indicated 100μm, (C) Bar indicated 500 μm,

(D) Bar indicated 100μm.

Supplemental Figure 3.

Neuropilin 1 was expressed in all cell types within the glomeruli.

Kidney specimens were co-stained against Neuropilin 1 (red) with specific markers for the three cellular components of glomeruli (green); Platelet endothelial cell adhesion molecule (PECAM) on endothelial cells (A), pococalyxin on podocytes (B) and alpha smooth muscle actin (ASMA) on mesangial cells (C). Bars, 20 μm.

Supplemental Figure 4.

Establishment of Sema3G knockout podocytes and wild-type podocytes

Glomeruli were isolated from mice and Sema3G deficient podocytes, as well as from

wild-type podocytes cultured from isolated glomeruli. The expression of podocyte-specific markers was evaluated by RT-PCR (A) and immunohistochemistry (B).

Bars indicated 25μm.

Supplemental Figure 5.

Sema3G inhibited the phosphorylation of p65 and ERK but not JNK and p38MAPK

Podocytes were pre-treated with 0.5 μg/ml Sema3G protein for 1 h and then stimulated with 0.1μg/ml of LPS for 30 min. Phosphorylation of p65, ERK, JNK, and p38MAPK was evaluated by Western blotting.

REFERENCE

1. Ruhrberg, C, Gerhardt, H, Golding, M, Watson, R, Ioannidou, S, Fujisawa, H, Betsholtz, C & Shima, DT: Spatially restricted patterning cues provided by heparin-binding VEGF-A control blood vessel branching morphogenesis. *Genes Dev* 2002; **16:** 2684-98.

Supplemental Figure 1

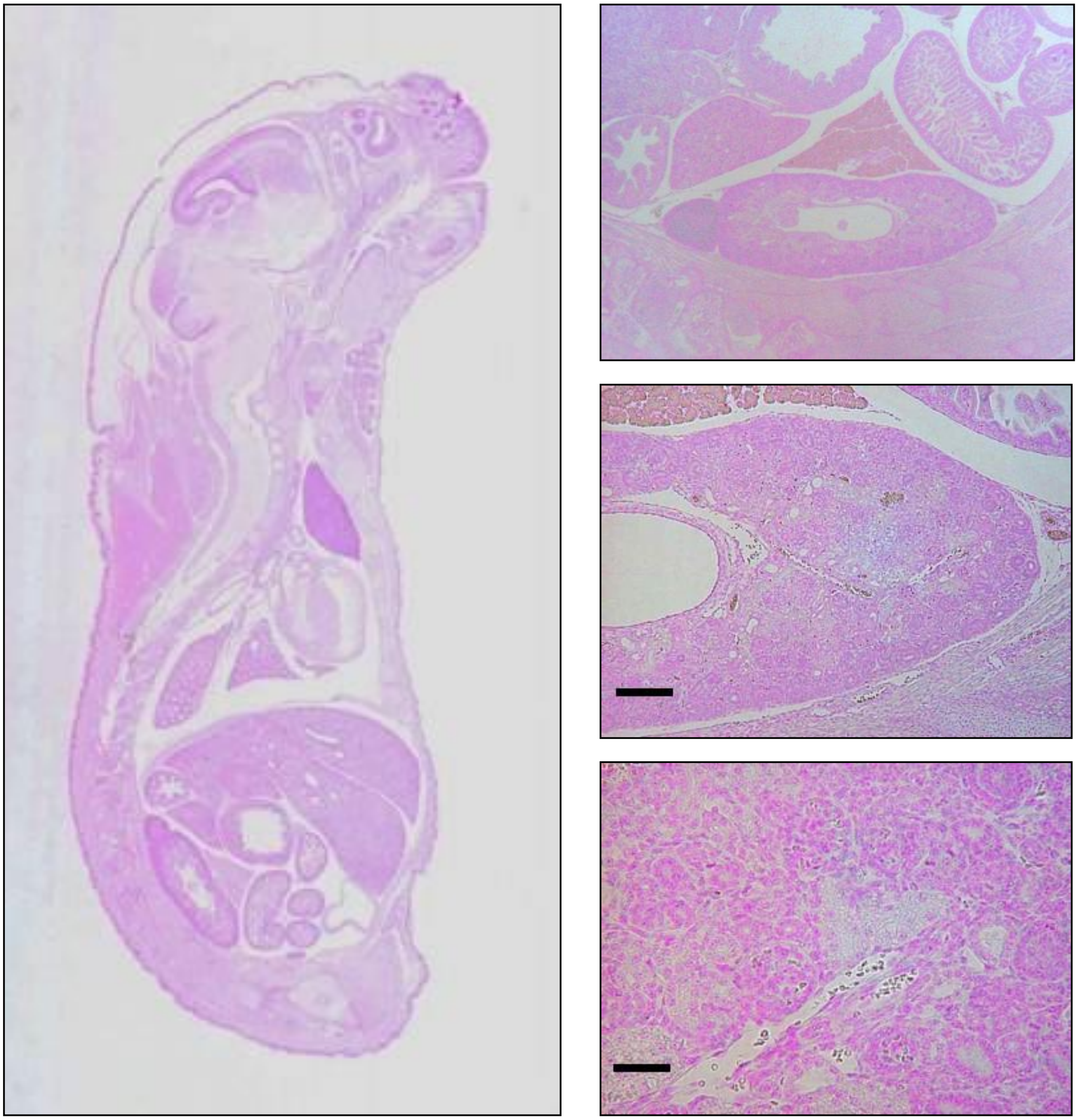

A novel podocyte gene, Semaphorin 3G, protects glomerular podocyte from lipopolysaccharide-induced inflammation

Ryoichi Ishibashi, Minoru Takemoto, Yoshihiro Akimoto, Takahiro Ishikawa, Peng He, Yoshiro Maezawa, Kenichi Sakamoto, Yuya Tsurutani, Shintaro Ide, Kana Ide, Harukiyo Kawamura, Kazuki Kobayashi, Hirotake Tokuyama, Karl Tryggvason, Christer Betsholtz and KoutaroYokote

Supplemental Figure 2

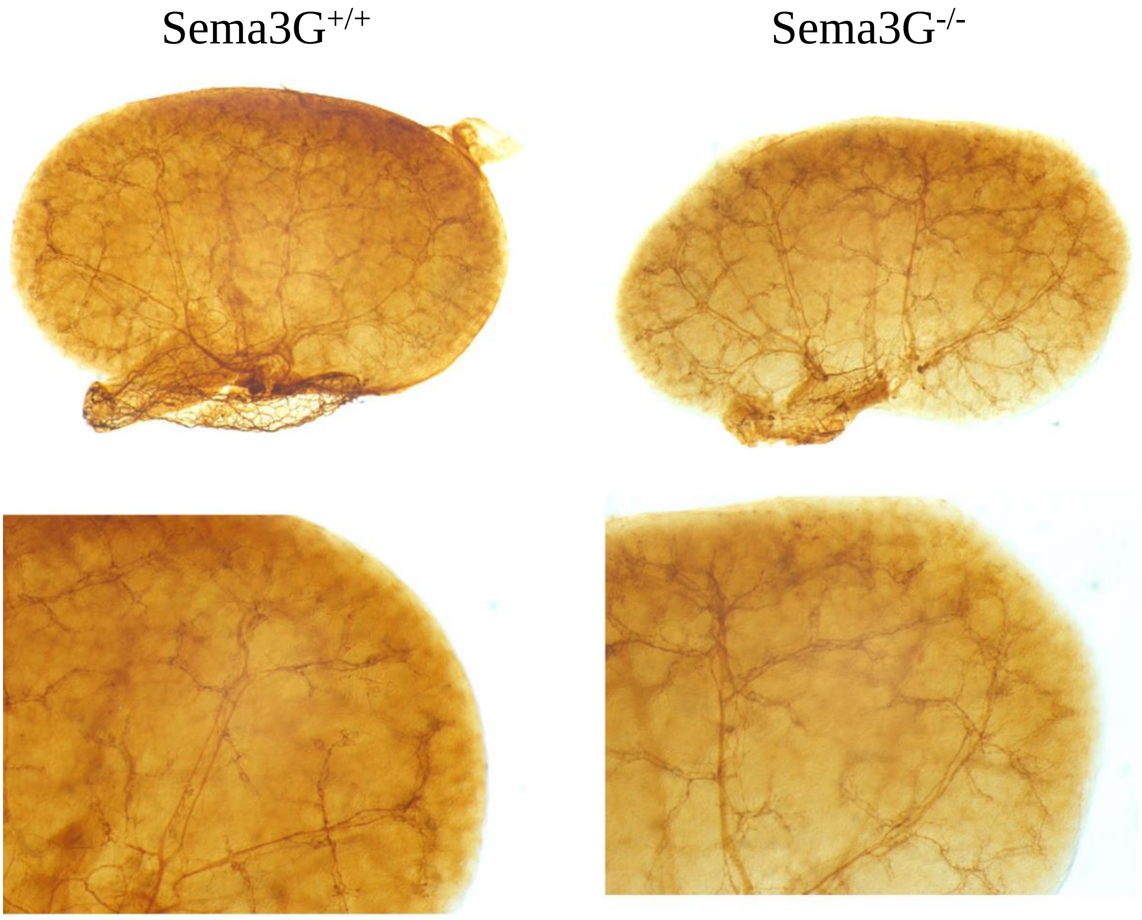

A novel podocyte gene, Semaphorin 3G, protects glomerular podocyte from lipopolysaccharide-induced inflammation

Ryoichi Ishibashi, Minoru Takemoto, Yoshihiro Akimoto, Takahiro Ishikawa, Peng He, Yoshiro Maezawa, Kenichi Sakamoto, Yuya Tsurutani, Shintaro Ide, Kana Ide, Harukiyo Kawamura, Kazuki Kobayashi, Hirotake Tokuyama, Karl Tryggvason, Christer Betsholtz and KoutaroYokote

Supplemental Figure 3

A

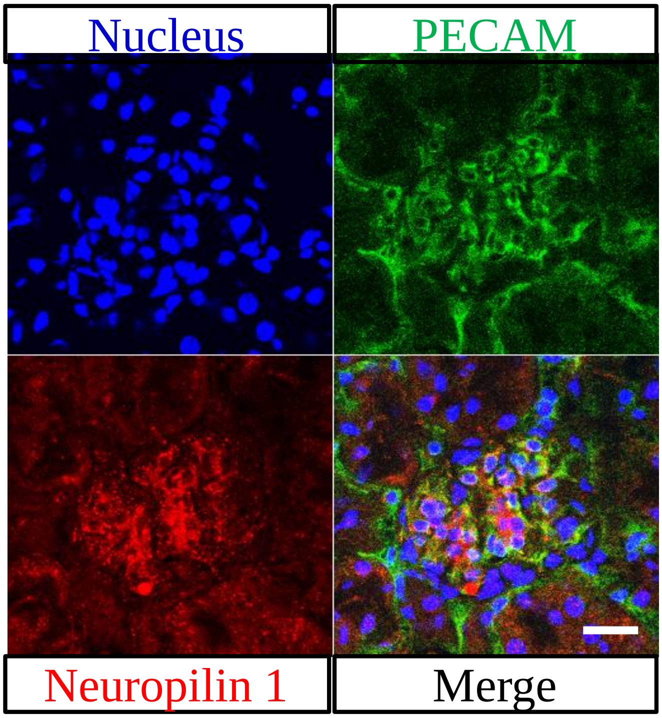

C

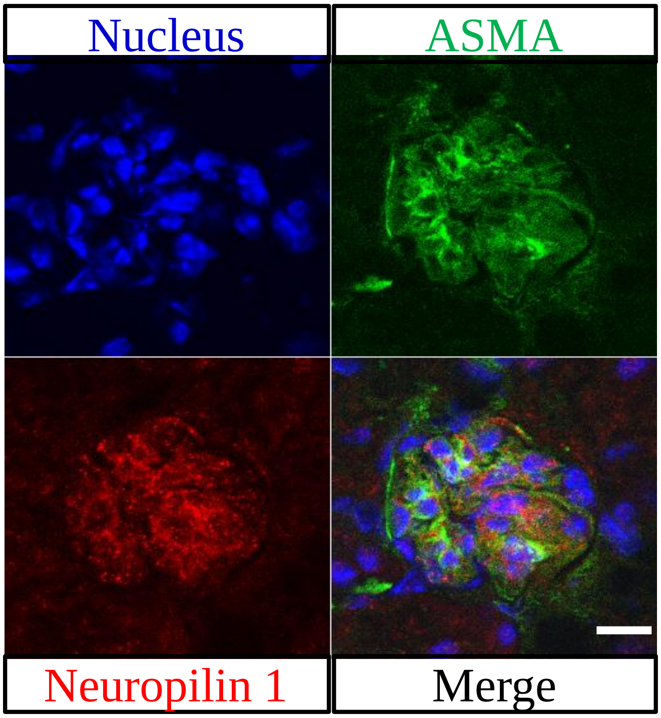

B

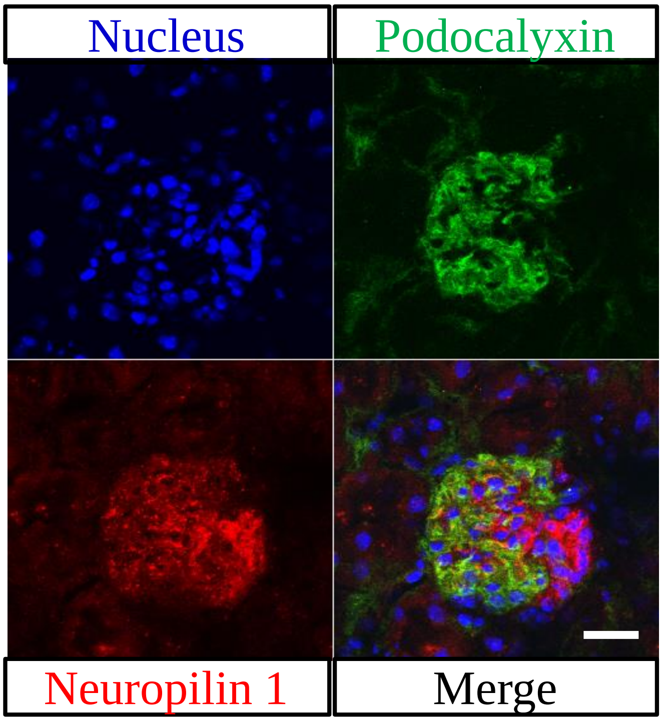

A novel podocyte gene, Semaphorin 3G, protects glomerular podocyte from lipopolysaccharide-induced inflammation

Ryoichi Ishibashi, Minoru Takemoto, Yoshihiro Akimoto, Takahiro Ishikawa, Peng He, Yoshiro Maezawa, Kenichi Sakamoto, Yuya Tsurutani, Shintaro Ide, Kana Ide, Harukiyo Kawamura, Kazuki Kobayashi, Hirotake Tokuyama, Karl Tryggvason, Christer Betsholtz and KoutaroYokote

Supplemental Figure 4

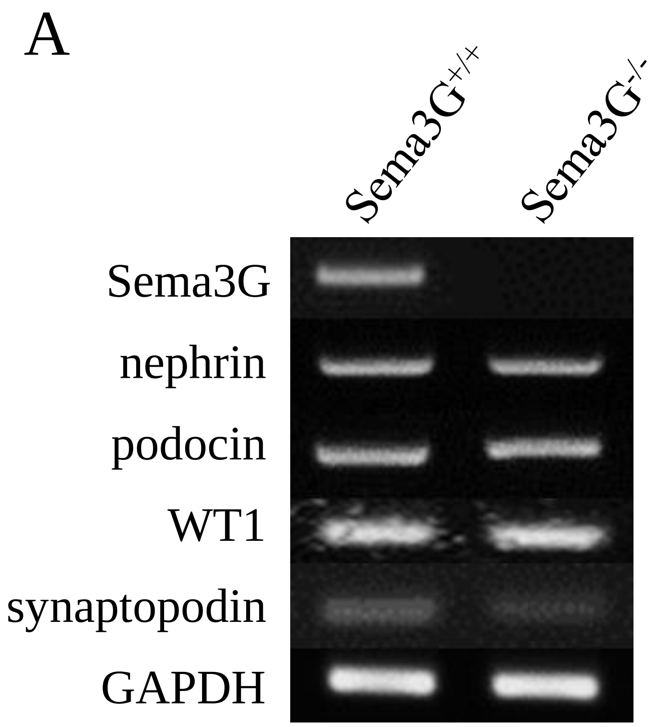

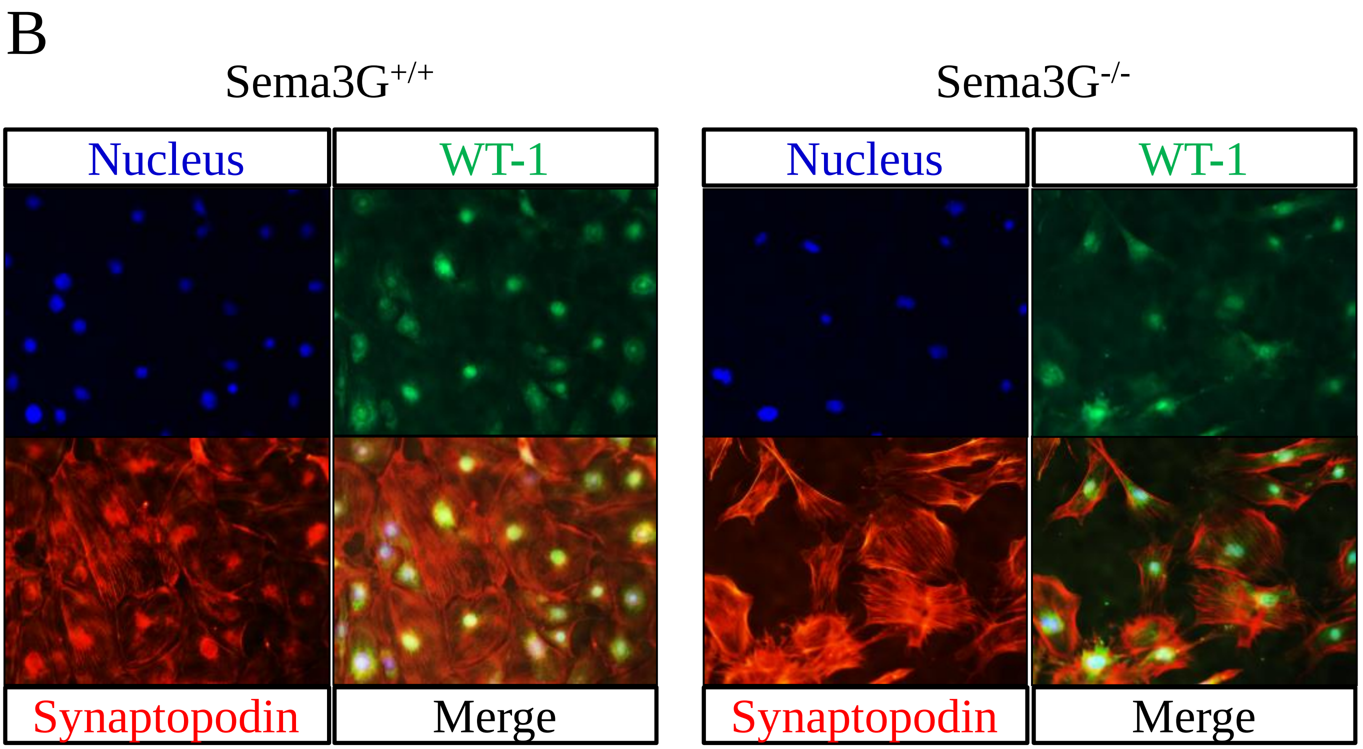

A novel podocyte gene, Semaphorin 3G, protects glomerular podocyte from lipopolysaccharide-induced inflammation

Ryoichi Ishibashi, Minoru Takemoto, Yoshihiro Akimoto, Takahiro Ishikawa, Peng He, Yoshiro Maezawa, Kenichi Sakamoto, Yuya Tsurutani, Shintaro Ide, Kana Ide, Harukiyo Kawamura, Kazuki Kobayashi, Hirotake Tokuyama, Karl Tryggvason, Christer Betsholtz and KoutaroYokote

Supplemental Figure 5

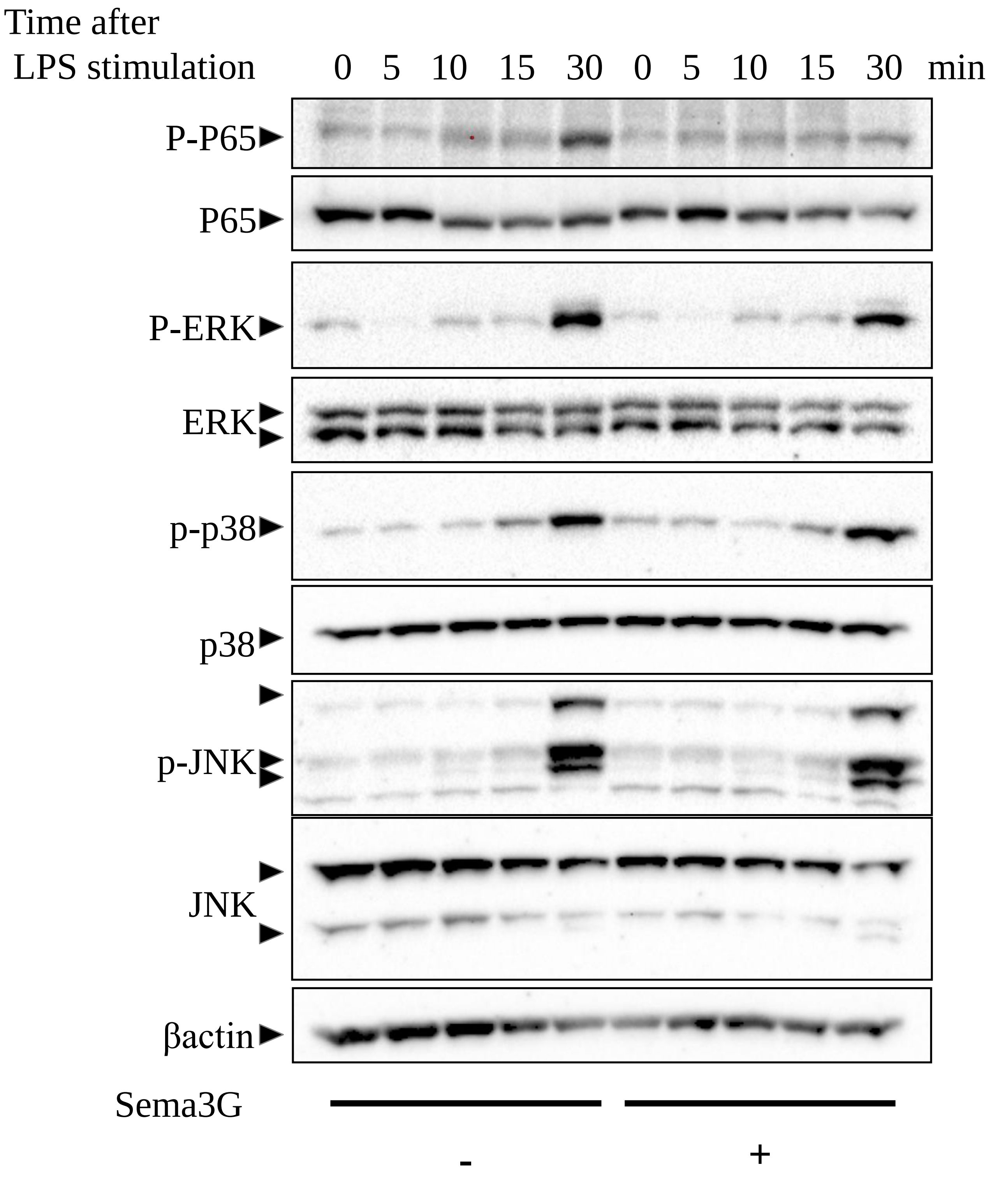

A novel podocyte gene, Semaphorin 3G, protects glomerular podocyte from lipopolysaccharide-induced inflammation

Ryoichi Ishibashi, Minoru Takemoto, Yoshihiro Akimoto, Takahiro Ishikawa, Peng He, Yoshiro Maezawa, Kenichi Sakamoto, Yuya Tsurutani, Shintaro Ide, Kana Ide, Harukiyo Kawamura, Kazuki Kobayashi, Hirotake Tokuyama, Karl Tryggvason, Christer Betsholtz and KoutaroYokote
